# Round about the Asylums. VIII

**Published:** 1888-06-23

**Authors:** 


### Round About the Asylums.—VIII.

By a Medical Superintendent.

### LINCOLN COUNTY ASYLUM.

Herein are 640 patients; and in addition 43 Lincoln cases are boarded out at the South Yorkshire Asylum, at a cost of 14*s.* a-week each. The weekly charge to the Lincolnshire unions is 8*s.* 9*d.*, and, as the visitors themselves remark, the ratepayers lose £600 a-year from the extra charge at Sheffield. The experience here is yet another proof, if any were needed, of the remark made in a previous notice, that when an asylum does not anticipate the wants of its county it always lags behind those wants. Dr. Palmer, after a long service, resigned the post of medical superintendent, and the visitors recommended him to Quarter Sessions for a pension of £600 a-year; but the visitors state they were disappointed in finding that their proposal was not supported in full, and the pension was cut down to £500. It is more than time that the clause relating to pensions in the Lunacy Acts was revised, and that medical superintendents were placed under some similar Act as are other Civil servants. Until then unseemly opposition will be sure to be made, and sometimes with success, as the above shows, while the visitors have the mortification of feeling that their careful decisions are not supported. The Commissioners in Lunacy pay a well-merited tribute to the manner in which Dr. Palmer managed the asylum. He was succeeded by Mr. Marsh. We wonder what the latter thinks of his chances of a pension. The farm is not a large one, and yet it is credited with a profit of £226. Judging from asylum reports, farming is a very profitable occupation, and we begin to think we have all lately expressed too much pity for the unfortunate farmer, who does not seem to be a very unfortunate man after all. The recoveries were 40 per cent. on the admissions, and the deaths were 14 per cent. on the average number resident; 166 patients were admitted during the year, and in not fewer than 89 of these the cause of insanity was hereditary influence. What a comment on our boasted civilisation! Well has it been said that man has been extremely careful in rearing his horses and cattle from healthy stock, but he thinks next to nothing about it when his own species is concerned.

### CARMARTHEN ASYLUM.

This is the asylum for Carmarthenshire, Cardiganshire, and Pembrokeshire. At the close of 1887 it contained 546 patients, 12 of these being of the private class. There are 47 vacant beds, and it is calculated these will be enough for the wants of the county for the next five years; 81 patients were admitted, and 80 were discharged, but the latter included 53 Glamorganshire cases. There were 39 deaths, and post-mortem examinations were made in 33 instances. The percentage of recoveries stands at 28·7, and the deaths at 7·68. Dr. Hearder attributes the low recovery-rate entirely to the duration of the insanity previous to admission, and he places the probably curable in the asylum as low as sixteen. Hereditary tendency accounts for 33 per cent. of the admissions, and intemperance in alcohol for 20 per cent. Dr. Hearder has completed his twentieth year in the service of the asylum, and he gives a brief *resumé* of its history. It seems the institution was built for 212 patients at a cost of £165 a bed, but this cost was reduced to £140 by certain modifications in the arrangements. It was added to in 1872, and again in 1875. In 1878 a mansion was rented not far from the asylum, and in 1879 the main building was once more enlarged, raising the total accommodation to nearly six hundred beds, at which it now stands. Dr. Hearder thinks that future legislation in lunacy matters will lead to an increase of private patients in county asylums. This is a very possible contingency, and it is undoubtedly a desirable one. The use of beer was discontinued eight years since, and the change has been "decidedly and absolutely beneficial." "The use of alcoholic stimulants in the treatment of disease, and as an addition to the diet of the feeble and aged, was gradually and almost completely discontinued. In this change also, there can be no doubt we made a decided advance in rational treatment." It would further seem that the mortality has decreased from 9·7 per cent. to 8·1 per cent. Whether this be owing to the disuse of alcohol or no it is almost impossible to say, but if so it would be in perfect harmony with other statistics; for instance, those of the United Kingdom Temperance and General Provident Institution, which show that the total abstainers enjoy a higher immunity from illness and death than the moderate drinkers who are insured in the same society. The weekly rate was 7*s.* 5½*d.* There are two assistant medical officers.

### STAFFORD COUNTY ASYLUM AT STAFFORD.

It is usually known as the "Old Stafford Asylum," to distinguish it from the other County Asylum at Lichfield. There were 826 patients resident at the end of the year, being an increase of fifty during the twelve months, and this increase was almost entirely confined to the female side of the asylum. The admissions were 257, the discharges 114, and the deaths 93. The chief causes of insanity in those admitted were hereditary tendency, domestic trouble, drink, and religion, accounting respectively for 38, 35, 19, and 12 of the cases. The recoveries were 29·5 per cent. on the admissions, and the deaths were 11·4 on the average number resident. The weekly cost is 8*s.*, and there are a few out-county cases received at 14*s.* a-week, but we do not notice any reference to private patients. The Commissioners' report is a somewhat long one, and its general tenor is favourable as to the condition of the asylum. Certain recommendations are made; for instance, additional means of exit from some of the dormitories, better supply of amusing literature, and an increase in the staff of attendants. We hear that the newly-adopted heating apparatus gave some trouble at first. It is curious to notice how often some forms of heating apparatus break down in asylums. The water supply is not very plentiful, a failing we have met with in many of the reports for 1887. Swine fever broke out at the farm, and fifty-two pigs were lost. In spite of this the farm produced a profit of £223, and it is only sixty-nine acres in extent. Early in the year Dr. Pater resigned the office of medical superintendent, and retired on his pension, but we regret to learn he died within a few weeks. He was succeeded by Dr. Christie.

### KILLARNEY DISTRICT ASYLUM.

This asylum contains 386 patients, being an increase of 18 on the previous year. The total number under treatment was 479, and in 37 per cent. of these the insanity was hereditary. Dr. Woods says he "must allow that at least in this county there has of late years been a decided increase of acute mania amongst the younger members of the population." There would seem to be one lunatic to 247 of the population in Limerick, and only one to 448 in Kerry, which is a strange and not easily accounted for difference. Dr. Woods adds: "As regards the sanity of the county, I would be glad to take these figures as correct, but I fear there are a large number of idiots and imbeciles either at home with their friends or wandering at large, not under proper care and control. As an example of this, we have the melancholy catastrophe which occurred near here within the last few days, when an entire family, six in number, all on the same day, suddenly developed acute mania, and during their excitement, an idiotic and epileptic member of the family lost his life." Calculated on the admissions, the recoveries were 34·2 per cent., and the deaths 12·2 on the average number resident. The weekly rate was 8*s.* 6*d.* The Inspector of Asylums states: "I have to express my very great satisfaction at the very creditable condition I found every part of the institution in."

